# In Situ Cutting of Ammonium Perchlorate Particles by Co‐Bipy “scalpel” for High Efficiency Thermal Decomposition

**DOI:** 10.1002/advs.202204109

**Published:** 2022-10-30

**Authors:** Peng Zhou, Siwei Zhang, Zhuoqun Ren, Xiaolin Tang, Kuan Zhang, Rui Zhou, Dan Wu, Jun Liao, Yifu Zhang, Chi Huang

**Affiliations:** ^1^ College of Chemistry and Molecular Sciences Wuhan University Wuhan 430072 China; ^2^ Research Center of Structure and Functional Materials Hubei Key Laboratory of Aerospace Power Advanced Technology Yichang 444200 China

**Keywords:** Coordination polymer, ammonium Perchlorate, catalytic mechanism, nano catalyst, structure self‐transformation

## Abstract

Burning rate of solid propellants can be effectively improved by adding catalysts and using smaller size ammonium perchlorate (AP). Although few reports, the exploration of changing the size of AP primary particles by catalysts is of great significance for improving combustion performance. Here, taking Co‐bipy as an example, the potential advantages of such materials as AP decomposition catalysts are reported. Due to the existence of NO_3_^−^ combined with oxygen rich environment provided by AP, the structural self‐transformation from micronrods to nanoparticles can be quickly realized during the heating process. More importantly, when Co‐bipy decomposes, it can play the role of “scalpel” and in situ cut AP particles. Results show that high‐temperature decomposition of Co‐bipy/AP occurs at 305.8 °C, which is 137.5 °C lower than that of pure AP. Catalytic mechanism is discussed by in situ IR and TG‐IR, CoO can effectively increase the content of reactive oxygen species and weaken the N–H bond, realizing the rapid oxidation of NH_3_. Eventually, the behavior of Co‐bipy cutting AP particles is tested. This interesting catalyst structure self‐transformation behavior can not only realize the influence on AP, but also perform a positive function in the combustion process of solid propellants, such as opening the adhesive AP interface.

## Graphical abstract

In this work, a simple synthesis strategy of an energetic material Co‐bipy is reported. In the presence of AP, Co‐bipy can rapidly realize the transformation from micro rod to nanoparticle. More importantly, when the Co‐bipy is decomposed, it can act as a "scalpel", In situ cutting of AP particles helps to improve the catalytic performance.

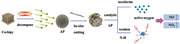

## 1 Introduction

As the power source of strategic missiles, the performance of solid propellants directly determines the combat effectiveness of weapons.^[^ ^1^, ^2^, ^3^ ^]^ Oxidants account for ≈70% of the propellant solid mass, and its decomposition behavior has a significant impact on the combustion of solid propellants. Since the 1940s, researchers have systematically discussed the thermal decomposition behavior of AP, the most common oxidant in solid propellants, and established its relationship with the burning rate by establishing a decomposition model. The results reveal that the faster decomposition of AP is more helpful to increase the burning rate.^[^ ^4^, ^5^, ^6^ ^]^ Additionally, the decomposition of AP is significantly affected by its own size and additives. The use of positive catalysts and the reduction of the original particle size of AP are considered to be the most effective means to change the thermal decomposition behavior of AP.^[^ ^7^, ^8^, ^9^, ^10^ ^]^

There are unsaturated points of the force field on the surface of the AP, such as defects and cracks, which are potential activation centers for the thermal decomposition of the AP. With the occurrence of decomposition behavior, these cracks and defects will continue to enlarge, resulting in the increase of internal stress, and finally the AP will be worn out into minor particles, the subsequent decomposition speed will be greatly accelerated.^[^ ^11^, ^12^, ^13^, ^14^, ^15^ ^]^ Chen explored the influence of NiO with different specific surface areas on the thermal decomposition behavior of AP. The results showed that the larger the specific surface area of NiO nanocrystals, the lower the high‐temperature decomposition peak of AP, and there was a certain functional relationship between the specific surface area and catalytic activity.^[^ ^16^ ^]^ Yi Wang prepared nano AP powder by ultra‐low temperature spray method and compared it with conventional AP powder. The test proved that nano AP has better thermal decomposition behavior, which is manifested in lower thermal decomposition temperature and richer gas products. On this basis, a feasible decomposition mechanism is proposed.^[^ ^17^ ^]^ Zhou explored the catalytic ability of composite metal oxide Co_3_O_4_/TiO_2_ for AP. The results showed that Co_3_O_4_/TiO_2_ could reduce the high‐temperature decomposition peak of AP to 295.0 °C. At the interface of Co_3_O_4_/TiO_2_ catalyst, enhancing the adsorption of NH_3_ through hydrogen bonding was considered to be the key to improving the catalytic effect.^[^ ^18^ ^]^ Although the small size AP can significantly reduce the decomposition temperature and increase the burning rate of propellant, its further application is limited by the safety questions in the preparation process (increased sensitivity due to collisions between small particles) and the special storage conditions required to prevent agglomeration. Most importantly, the use of a small‐sized AP will lead to a passive adjustment of other components in the propellant, which will pose a great challenge to the overall design of the propellant.^[^ ^17^ ^]^ Therefore, it is of great significance to develop high‐burning rate solid propellants that how to refine the AP particle size in situ during combustion by adding catalyst without changing the original propellant formula. At the same time, in butanediol/polyurethane propellant and other systems, AP particles are wrapped by adhesives, which will affect the combustion behavior of propellant to a certain extent, therefore, how to open the combustion channel through catalyst will be interesting work.

Metal complexes (MCP) were once taken into account as the best choice of catalysts in various research fields since their dispersed active sites, simple synthesis conditions, and efficient catalytic behavior.^[^ ^19^, ^20^, ^21^, ^22^ ^]^ In addition, metal/metal clusters and ligands can be reasonably optimized and functionally designed according to different application scenarios, that is, the adjustable behavior of MCP endows them with the ability to pursue more efficient catalysis.^[^ ^23^, ^24^, ^25^ ^]^ For the thermal decomposition reaction of AP, introducing energetic components (such as NO_3_^−^ and ClO_4_^−^) into the structure of MCP can change their decomposition behavior, producing the effect of high‐energy “explosives” like RDX and HMX, which can not only reduce the size of catalytic active center, but also significantly improve the agglomeration behavior of catalysts. However, the good thermal stability of MCP makes it difficult to realize such ideas. Based on this, researchers try to induce the structural transformation of MCP through media to release active centers more quickly. As an example, Deng et al. used MOF as the metal precursor and laser as the light source to irradiate MOF crystal through a nanosecond pulse laser, successfully destroying the MOF coordination structure and rapidly obtaining metal nanoparticles.^[^ ^26^, ^27^, ^28^, ^29^, ^30^ ^]^ Fortunately, in the thermal decomposition of catalytic AP, MCP can not only be used as catalysts for the decomposition of AP, but also be used as “catalysts” for the decomposition of MCP, because it creates an oxygen rich environment, which is conducive to the conversion of MCP from M‐N bond to M‐O bond. Based on the inspiration of the structural characteristics of MCP, this study accelerated the mutual catalysis of AP and MCP in the decomposition process by selecting appropriate metal active centers and introducing energetic components into the complex structure. The influence of AP on the thermal stability of MCP and the “cutting” behavior of metal complexes on AP were verified by scanning electron microscopy (SEM) and differential scanning calorimetry (DSC) respectively.

This study takes Co‐bipy by example to reveal the potential advantages of this kind of energetic materials as catalysts for thermal decomposition of AP. Benefiting from the oxygen rich environment provided by AP, Co‐bipy will decompose in advance to produce CoO nanoparticles with high‐speed impact force, cutting adjacent AP particles, which can reduce the size of AP in situ while achieving good dispersion of CoO. The physicochemical properties and thermal decomposition products of Co‐bipy were characterized by XRD, SEM, TEM, BET, and XPS. The thermal decomposition effect of Co‐bipy catalyzed AP was analyzed by TG and DSC. On this basis, the structure transformation and real‐time decomposition products of Co‐bipy/AP were studied by TG‐IR and in situ infrared spectroscopy, and its catalytic mechanism was proposed. The potential applications of this kind of materials and the future design direction of catalysts in solid propellants are discussed in Outlook part.

## 2 Result and Discussion

**Figure 1:**
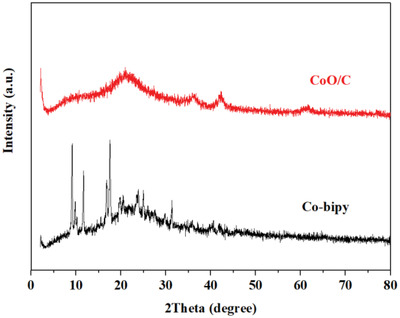
XRD diffraction patterns of Co‐bipy and CoO/C.

Using 4, 4‐bipyridine (bipy) as the organic ligand, the Co^2+^ coordinated Co‐bipy material was prepared by solvent synthesis method.^[^ ^31^ ^]^ The reaction was carried out through the initial nucleation of small MCP crystals and the self‐assembly of bridged Co clusters and NO_3_^−^and 4,4‐bipyridine in the coordination structure (Figure S7, Supporting Information).^[^ ^31^ ^]^ The X‐ray diffraction (XRD) results of Co‐bipy materials before and after heat treatment are shown in **Figure** **1**. The experimental XRD spectra are imported into diamond software. By comparing with the results of the standard spectra, the matching rate is more than 95%. Due to the characteristics of coordination structure, the metal sites are dispersed in the coordination network, which enhances the utilization of catalytic sites of Co‐bipy materials and helps to improve the thermal decomposition performance of catalytic AP. The XRD spectrum of Co bipy after heat treatment at 400 °C proves that CoO/C is the final product after pyrolysis.

**Figure 2:**
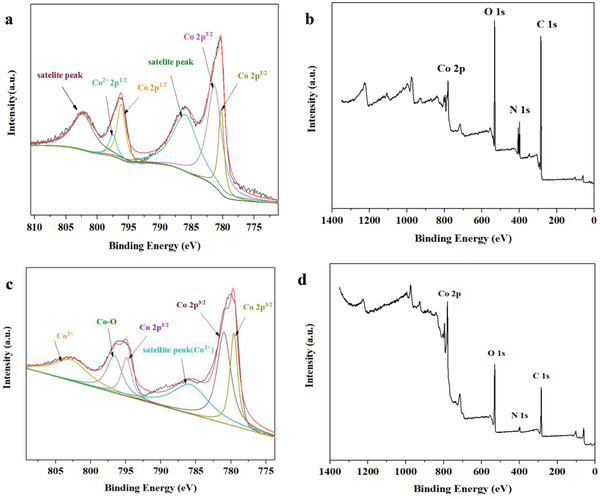
a) Co 2p and b) XPS spectra of Co‐bipy; c) Co 2p and d) XPS spectra of CoO/C.

The elemental composition and valence states of the surfaces of Co‐bipy and thermal decomposition product were obtained by XPS (**Table** **1**). The content of Co on the surface of Co‐bipy was only 2.56%, but increased to 9.23% after heat treatment, which showed an obvious increasing trend and was caused by the destruction of coordination structure and the formation of partial gaseous products under high‐temperature pyrolysis. More importantly, ≈52% C content and a certain amount of N were observed in the decomposition products of Co‐bipy, which proved that the calcined products of Co‐bipy were CoO/N‐doped carbon (hereinafter referred to as CoO/C), and the CoO as the catalytic main body was coated with C (the structural characteristics of Co by coordination structure), thus avoiding the agglomeration behavior of active sites, based on this, due to the principle of XPS testing, the actual content of Co in CoO/C may be more. In addition, the XPS test results of Co‐bipy and CoO/C are shown in **Figure** **2**. For Co‐bipy, the characteristic peaks of C, N, O, and Co appear at 285, 、406, 、532, and 781 eV respectively, of which the characteristic peaks at 796.2 and 797.6 eV correspond to Co 2p^1/2[^ ^32^, ^33^ ^]^; The characteristic peaks at 780.2 and 781.4 eV correspond to Co 2p^3/2[^ ^34^, ^35^ ^]^; The characteristic peaks at 802.4 and 786 eV correspond to the satellite peaks of Co.^[^ ^36^, ^37^ ^]^ In contrast, the characteristic peaks of C, N, O, and Co of CoO/C appear at 285, 、399, 、531, and 780 eV respectively. For Co, the characteristic peaks at 779.6, 781, and 794.9 eV correspond to Co 2p^3/2^,^[^ ^38^, ^39^, ^40^ ^]^ and the characteristic peaks at 803.1 eV correspond to Co^2+[^ ^41^ ^]^; The characteristic peak at 796.6 eV corresponds to Co–O.^[^ ^42^ ^]^ in addition, the characteristic peak at 785.9 eV corresponds to the satellite peak of Co^2+^ .^[^ ^43^ ^]^

**Table 1:** Chemical compositions of Co‐bipy and CoO/C as determined by XPS

Samples	C (mass%)	N (mass%)	O (mass%)	Co (mass%)
Co‐bipy	47.86	29.83	19.74	2.56
CoO/C	52.09	23.29	15.39	9.23

**Figure 3:**
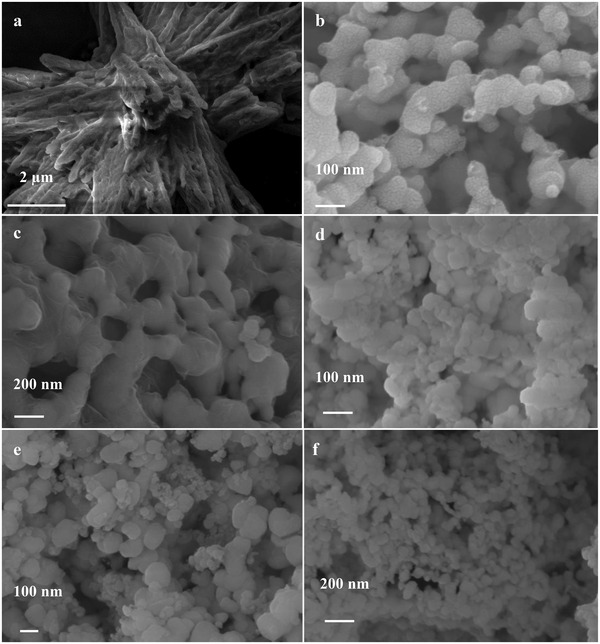
a) SEM spectra of Co‐bipy; b) CoO/C (1 °C min^−1^); c) CoO/C (2 °C min^−1^); d) CoO/C (5 °C min^−1^); e) CoO/C (10 °C min^−1^) and f) CoO/C (20 °C min^−1^).

SEM revealed the structural transformation of Co‐bipy under heat treatment. The test results are shown in **Figure** **3**. The initial Co‐bipy is an assembly of micron rods, and has obvious agglomeration behavior; in contrast, the structure of CoO/C treated at different heating rates is obviously different from that of Co‐bipy. It exists in the form of 100–200 nm nanoparticles. For the combustion reaction of solid propellant, the system can reach an extremely high temperature in a very transitory time. Therefore, for the experimental simulation of the heat treatment process, the faster the heating rate, the closer to the real scene. The size changes of CoO/C at different heating rates were investigated. The results showed that with the increase of heating rate, the size of nanoparticles almost did not change significantly, which was due to the structural characteristics of Co‐bipy. In the heat treatment state, Co‐bipy decomposed, and the presence of NO_3_^−^ led to the “explosion” of agglomerated nanoparticle assemblies, which evolved into dispersed nanoparticles. More importantly, when AP and Co‐bipy are fully mixed, due to the “explosive decomposition” characteristics of Co‐bipy, the huge impact force generated by Co‐bipy can realize in situ cutting of AP particles, resulting in more cracks and defects on the surface of AP particles, and even breaking AP into smaller structures, which will significantly increase the “active sites” at the initial stage of AP decomposition, thus significantly changing the thermal decomposition behavior of AP. As a comparison, different cobalt precursors (cobalt chloride/cobalt acetate, hereinafter referred to as Co‐bipy‐2 and Co‐bipy‐3) are used to obtain corresponding products under the same reaction conditions and the same ligand precursor, and their structure self‐transformation behavior was explored. The results are shown in Figure S1, Supporting Information. Taking Co‐bipy‐2 as an example, although high‐temperature heat treatment causes its decomposition behavior (Figure S6, Supporting Information), by observing its microstructure, no morphological transformation behavior before and after thermal decomposition is found. Compared with the results of Co‐bipy under the same heat treatment conditions, which proves that NO_3_^−^ is an important reason for the self‐transformation of Co‐bipy.

**Figure 4:**
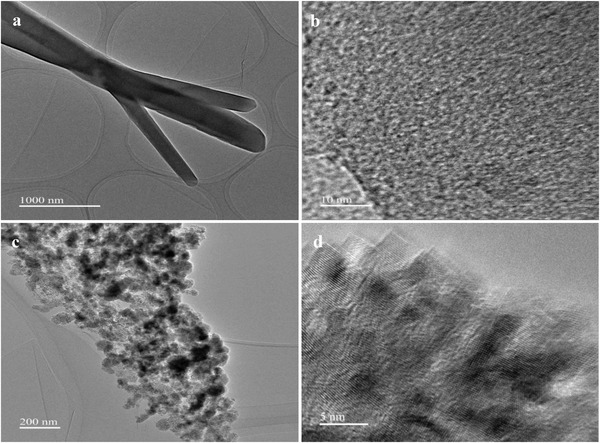
a‐b) TEM spectra of Co‐bipy and c‐d) CoO/C.

The structural transformation of Co‐bipy before and after heat treatment was further determined by TEM. The test results are shown in **Figure** **4**. Co‐bipy shows a solid rod structure, which is consistent with the SEM test results. The existence of lattice stripes is not detected after magnification; it is observed in Figure 4c that the CoO/C size after heat treatment is basically the same, all of which are nanoparticles of ≈100 nm. By observing the independent nanoparticles (Figure S2, Supporting Information), the uniform pore structure is found, referring to the relevant data of the BET test, it is further confirmed that high‐temperature heat treatment can not only realize nanostructure, but also multi‐porosity (**Table** **2**). Furthermore, the existence of CoO was confirmed by measuring the spacing between adjacent lattices. The measured lattice spacing was 0.25 nm, corresponding to CoO (111) crystal plane.^[^ ^44^, ^45^ ^]^ Nanostructures combined with porous characteristics are helpful to release more catalytic active sites and accelerate electron transfer in the catalytic process. Combined with the good advantages of CoO itself in the catalytic thermal decomposition of AP, Co‐bipy is expected to become a new generation of functional AP thermal decomposition catalyst.

**Table 2:** Pore characteristics of samples

Sample	Co‐bipy	CoO/C
*S*_BET_ (m^2^ g^−1^)	121.34	466.16
*S*_micro_ (m^2^ g^−1^)	39.90	205.93
*V*_total_ (nm)	0.05	0.23
*W*_aver_(nm)	0.79	0.75

**Figure 5:**
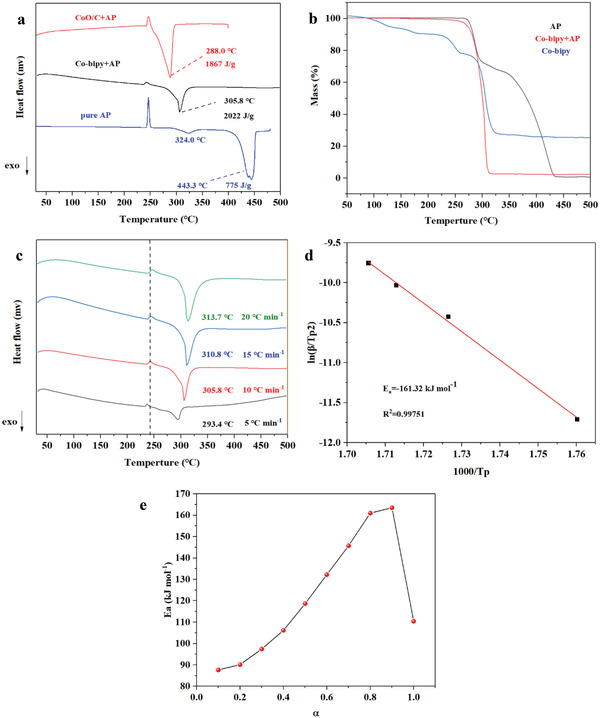
a) DSC curves of Co‐bipy and CoO/C catalyzed thermal decomposition of AP; b) TG curve of Co‐bipy/AP; c) DSC curve of d) Co‐bipy/AP; Ea linear fitting result of e) Co‐bipy/AP at different heating rates and change trend of reaction activation energy with reaction progress.

Based on the analysis of the microstructure and chemical composition of Co‐bipy and CoO/C, the catalytic thermal decomposition performance of Co‐bipy and CoO/C for AP was discussed. The test results are shown in **Figure** **5**. For pure AP, an obvious characteristic peak of crystal transformation is observed at 246.1 °C. With the increase of temperature, the first exothermic peak emerges near 324.0 °C. At this time, corresponding to the low‐temperature decomposition stage of AP, the proton transfer from NH_4_^+^ to ClO_4_^−^ commence, followed by the generation of NH_3_ and coverage on the AP surface. At 347.7 °C, the low‐temperature decomposition stage stops, and the mass loss in this process is relatively small. With the further increase of the system temperature, it enters the high‐temperature decomposition stage near 443.3 °C. The desorption of NH_3_ and releases a great number of AP active interfaces as the starting sign. With the accomplished decomposition of AP, a series of gas‐phase products are generated, showing a large number of exothermic phenomena. Co‐bipy and CoO/C showed good effects on reducing the high‐temperature decomposition peak of AP and changing the thermal decomposition behavior of AP. They can reduce the high‐temperature decomposition peak of AP to 305.8 °C and 288.8 °C respectively. At the same time, the overall process is accompanied by 2022 J g^−1^ and 1867 J g^−1^ energy release. Compared with 775 J g^−1^ of pure AP, the energy release level in the course of the thermal decomposition of AP is significantly improved. It is worth mentioning that, in comparison with the LTD‐HTD behavior of pure AP, the thermal decomposition behavior of AP has completely changed under the catalysis of Co‐bipy and CoO/C, and no characteristic low‐temperature decomposition stage has been observed in the whole decomposition process. This is because Co‐bipy and CoO/C have good catalytic activity, which can change the thermal decomposition behavior of AP by promoting the proton/electron transfer process and accelerating the decomposition of HClO_4_ (contributing more active oxygen and accelerating the oxidation of NH_3_). It can also be seen from the TG results that Co‐bipy/AP is completely decomposed within the low‐temperature decomposition range corresponding to AP. In addition, the role of Co‐bipy as a “scalpel” was further verified. Ammonium nitrate (explosion behavior during decomposition) was used as the “catalyst” for thermal decomposition of AP for DSC test (Figure S3, Supporting Information). The results showed that the high‐temperature decomposition peak of AP still decreased by 37 °C without the presence of catalytic active center in ammonium nitrate, which proved that reducing the particle size of AP could effectively improve its decomposition efficiency. In addition, the average activation energy of Co‐bipy catalyzed thermal decomposition of AP was further calculated by using the Kissenger equation (Figure 5d). Compared with the 328.9 kJ mol^−1^ activation energy of pure AP, Co‐bipy can significantly reduce it to 161.3 kJ mol^−1^, which proves that the thermal stability of AP is significantly affected in the presence of Co‐bipy. For catalytic AP thermal decomposition, the lower decomposition activation energy is more conducive to the next stage of propellant combustion, so as to improve the burning rate of propellant. In addition, the activation energy of Co‐bipy/AP under different reaction processes is calculated by Ozawa method. The calculation results are shown in Figure 5d. The whole process is consistent with the thermal decomposition behavior of Co‐bipy/AP. Although the activation energy reaches the maximum value (163.32 kJ mol^−1^) at 90% of the reaction process, it still proves the good catalytic ability of Co‐bipy and has excellent catalytic prospects.

## 3 Catalytic Mechanism

**Figure 6:**
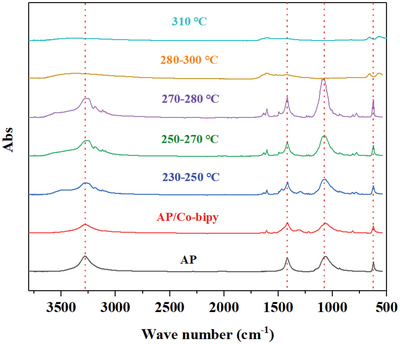
In situ infrared test results of Co‐bipy/AP.

In situ characterization can provide real‐time structural and decomposition behavior changes of Co‐bipy/AP. Based on this, the thermal decomposition mechanism of Co‐bipy catalyzed AP can be further obtained. The structure change of Co‐bipy/AP with temperature was monitored by in situ infrared test. The test results are shown in **Figure** **6**. From the test results, it can be found that for the initial AP sample, the stretching vibration peak of ClO_4_^−^ is located at 619 cm^−1^ and 1030 cm^−1^, while the bending vibration peak and stretching vibration peak of N–H bond are located at 1410 cm^−1^ and 3270 cm^−1^ respectively. In addition, the characteristic peaks at 1600 cm^−1^ and 781 cm^−1^ correspond to the characteristic peaks of ligands in Co‐bipy. When the system temperature is at 230—280 °C, the vibration peak of the Co‐bipy/AP mixed sample does not change, which proves that there is no decomposition behavior of AP and Co‐bipy at this time. With the increase of reaction temperature, the intrinsic characteristic peaks of AP and Co‐bipy cannot be observed at 280—300 °C. this significant change indicates that Co‐bipy is gradually adsorbed by AP. At this time, the decomposition reaction of Co‐bipy and AP is carried out at the same time; when the heat treatment temperature increases to 310 °C, the characteristic peak of AP has been basically not observed, and two characteristic peaks of Co–O gradually appear near 600 cm^−1^, which proves that the coordination structure of Co–N bond in the original Co‐bipy has been gradually replaced by Co–O bond, and also indicates that the thermal stability of Co‐bipy will be greatly reduced in the presence of AP. The weakening of Co‐N bond in Co‐bipy coordination structure is caused by the rapid decomposition of Co‐bipy and the formation of Co–O bond by the chemisorption of Co atom on the surface of Co‐bipy and O atom on the surface of AP, that is, NO_3_^−^ and AP jointly catalyze the rapid transition from Co‐bipy to CoO. The electronegativity of the O atom is 3.44, which is greater than 3.04 of N atom. The ability of O atom to attract electrons is stronger than that of N atom. The electron cloud between Co‐N bonds will shift to the direction of Co–O bond, that is, the Co–O bond weakens the Co‐N bond. To summarize, in situ infrared testing has proved the transformation behavior of Co‐bipy to CoO in the presence of AP. Based on this, it is speculated that the thermal decomposition of AP catalyzed by Co‐bipy is mainly divided into two stages. First, in the presence of AP, Co‐bipy is thermally decomposed and transformed into CoO/C with catalytic activity. There is one more point, the generated CoO/C catalyzes the decomposition of AP, and finally changes the decomposition behavior of AP.

**Figure 7:**
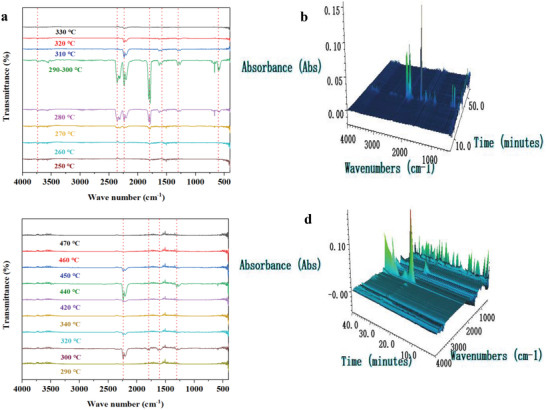
a,b) Co‐bipy/AP and c,d) AP process in situ gas production.

Although the structural transformation of Co‐bipy in the catalytic process was detected by in situ infrared spectroscopy, the specific reason and process for its change in AP decomposition behavior are not clear. In the process of AP catalytic thermal decomposition, intermediate products NH_3_ and HClO_4_ will be produced, the stronger the catalytic activity of the catalyst, the faster the decomposition of HClO_4_ and the higher the concentration of active oxygen generated.^[^ ^46^, ^47^ ^]^ Subsequently, NH_3_ will be oxidized more thoroughly, and the valence of nitrogen in the nitrogen‐containing oxides generated will be higher. Therefore, analyzing the composition of nitrogen‐containing oxides (including N_2_O, NO, and NO_2_) produced by the thermal decomposition of AP can judge the catalytic activity of the catalyst, so as to better understand the catalytic reaction mechanism. The gas phase products in the course of the thermal decomposition of AP catalyzed by Co‐bipy were monitored by TG‐IR. As a comparison, the in situ gas products of pure AP were tested. The results are shown in **Figure** **7**. For pure AP, the monitored gas product first appears at ≈300 °C, the main characteristic peak position is 2300 cm^−1^, and the corresponding gas product is N_2_O. With the increase of reaction temperature, the peak intensity of N_2_O increases gradually, then decreases until it disappears temporarily, which corresponds to the completion of the low‐temperature decomposition stage of AP. With the progress of monitoring time, the high‐temperature decomposition stage of AP begins, accompany the emergence of NO and NO_2_ characteristic peaks, the maximum value is reached at ≈440 °C, and no gas signal is detected at ≈450 °C, which proves that AP has been completely decomposed at this time (corresponding to the temperature of 460 °C). According to the whole AP decomposition process, N_2_O, NO, and NO_2_ are the main gaseous products of AP thermal decomposition. In contrast, Co‐bipy catalyzes the thermal decomposition of AP, showing completely different gas generation behaviors. It not only has typical AP decomposition characteristic gas products, but also has a gas characteristic peak of CO_2_, which corresponds to the decomposition behavior of Co‐bipy catalyst in this temperature range (270–300 °C). Compared with the thermal decomposition characteristics of pure Co‐bipy, the presence of AP creates an oxygen rich environment and accelerates the production of CoO. Therefore, for Co‐bipy, the actual catalytic component is not the MCP itself, but its thermal decomposition product. The main thermal decomposition behavior of Co‐bipy/AP occurs in the range of 270–330 °C. Different from pure AP, NO_2_ is the main gas phase decomposition product, and the content of nitrogen oxides in the high valence state is significantly increased, which proves that the energy required for NH_3_ to be oxidized to the nitrogen‐containing gas products in the high valence state can be provided in the reaction system, that is, a higher concentration of reactive oxygen species.

**Figure 8:**
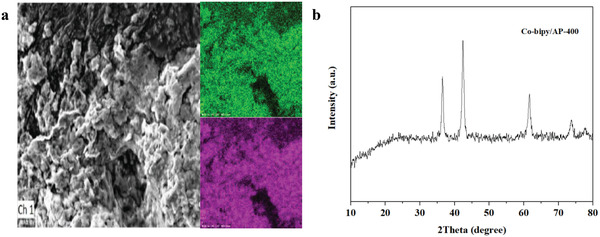
a) SEM and EDS test and b) XRD test results of Co‐bipy/AP‐400.

The residual substance after Co‐bipy/AP heat treatment was characterized by EDS and XRD to accurately obtain the final structure and morphology of the catalyst. The test results are shown in **Figure** **8**. SEM represents that the size of nanoparticles does not change significantly after heat treatment, but there is an obvious agglomeration phenomenon. This is because a large amount of vapor phase substance will be produced during the thermal decomposition of AP. For the reason that the catalyst is well dispersed in the AP system, the formation of vapor phase products will take away the catalyst powder, which will secondarily be deposited on the magnetic boat through vapor deposition, therefore, the final behavior is agglomeration. In addition, the content ratio of Co and O in EDS test results is close to 1:1. According to the XRD test results in Figure 8b, after the catalytic reaction, the catalyst still exists in the form of CoO. Although it has experienced a strong oxidation atmosphere, it still has good catalytic stability.

**Figure 9:**
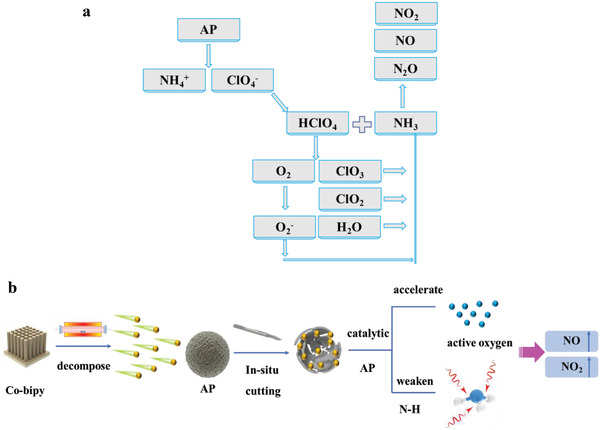
a) AP thermal decomposition process and b) Co‐bipy catalyzed AP thermal decomposition mechanism.

To sum up, the mechanism of Co‐bipy catalytic thermal decomposition of AP is as follows: First, in the presence of AP, Co‐bipy will be thermally decomposed and transformed into a CoO catalyst with catalytic activity. At the same time, with the formation of nanometer catalyst structure, the agglomeration phenomenon is obviously resolved. By accelerating the proton transfer process and the decomposition of HClO_4_, the generated CoO rapidly increases the concentration of active oxygen in the system, and then rapidly oxidizes the NH_3_ generated in the low‐temperature decomposition stage of AP to avoid it being covered on AP surface, thus shielding the AP deactivation stage between LTD‐HTD during the decomposition process of AP. Specifically, it combines the thermal decomposition of the whole AP into a characteristic peak. In addition, due to the good catalytic activity of CoO and the fact that the gas characteristic peak of NH_3_ was not detected in the TG‐IR test, it proves that coo had a good ability to weaken the N–H bond. Combined with a large number of active oxygen in the system, the oxidation degree of N was significantly increased during the decomposition of AP, the corresponding NO_2_ was significantly generated, and the heat release of the system was significantly increased (**Figure** **9**).

## 4 Co‐Bipy Cutting AP Behavior

**Figure 10:**
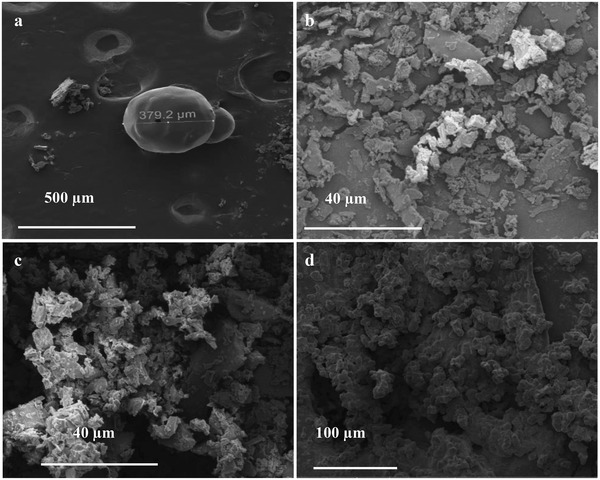
a) SEM spectra of AP; b) Co‐bipy/AP‐280; c) Co‐bipy/AP‐290 and d) Co‐bipy/AP‐300.

Based on the discussion of catalyst structure and catalytic mechanism, the “scissors” effect of Co‐bipy in the process of catalytic AP was further testified by SEM testing of Co‐bipy/AP at different heat treatment temperatures. As the decomposition of AP under catalysis is continuous and high‐speed, the mixed sample is rapidly cooled at high temperature to obtain the instantaneous product state. Considering the time brought by this operation, this result is only used to judge the behavior of Co‐bipy cutting AP. **Figure** **10**a shows the initial AP particles with a size of ≈380 µm. when the heat treatment temperature reaches 280 °C (Figure 10b), the Co‐bipy/AP shows an irregular fragment structure with a size of 5–40 µm, at this time, it is corresponding with the decomposition of the Co‐bipy. Combined with the above discussion on the catalytic process, it is speculated that the fragmentation of the AP at this time is due to the cutting behavior generated by the decomposition of the Co‐bipy, which makes the AP into smaller irregular fragments. As the reaction process progresses, AP has no obvious size and shape at 290 °C (Figure 10c), which proves that complete decomposition of AP has not occurred at this time. When the reaction temperature reaches 300 °C (Figure 10d), the structure of AP changes again and becomes smaller AP particles, which corresponds to the “local chemistry” behavior of AP decomposition. The smaller AP particles accelerate its thermal decomposition rate. In general, Co‐bipy cuts AP into smaller irregular structures through its own decomposition behavior to provide more decomposition active centers, so that AP can undergo small particle structure transformation at lower temperatures, thus significantly reducing the decomposition peak temperature of AP and increasing the decomposition rate of AP.

## 5 Conclusion

In conclusion, taking Co‐bipy as an example, we proved the structure evolution of the catalyst in the process of catalytic AP thermal decomposition and observed the behavior of Co‐bipy cutting AP particles. On this basis, the mechanism of catalytic AP thermal decomposition was discussed. The results demonstrate that Co‐bipy acts on AP following the process of decomposition before catalysis. The high‐temperature decomposition stage of Co‐bipy/AP occurs at 305.8 °C, which is 137.5 °C lower than that of pure AP. In addition, it also contributes to the exothermic of 2022 J g^−1^. The excellent catalytic effect not only depends on the in situ size transition during Co‐bipy decomposition, but also changes from micron rod structure to well‐dispersed nanoparticles; at the same time, due to the in situ cleavage of AP particles during Co‐bipy decomposition, AP is fragmented to expose more active sites, the combined action of the two results in a better catalytic effect. Finally, the thermal decomposition mechanism of AP catalyzed by Co‐bipy was explored. The results showed that CoO could effectively weaken the N–H bond, accelerate the rapid decomposition of HClO_4_, contribute more active oxygen to the system, accelerate the oxidation of NH_3_, and rapidly decompose AP. This study provides a strategy for the catalyst to impact the AP structure in situ, which is helpful to understand the catalytic decomposition process of AP and develop more efficient combustion catalysts.

## 6 Perspective

We have reported the research on the structural transformation of Co‐bipy after heating to achieve in situ cutting of AP particles. There is no doubt that this functional combustion catalyst gives a variety of possibilities to improve the burning rate compared with traditional materials. The advantage of Co‐bipy is the effective dispersion of catalytic sites and the destruction of AP particles. Combined with the functionality of energetic components, it contributes to better catalytic behavior on the basis of improving the energy level of solid propellants. Despite the remarkable results of the first attempt, the exploration and practical application of such functional combustion catalysts still face challenges, including how to achieve more metal content on the basis of ensuring structural functionality. Some future research directions and operable solutions are also proposed to overcome the challenges at this stage, as follows:

1) The stability of Co‐bipy for burning rate increase needs to be further determined. Although the burning rate test is not shown in this manuscript, the irregularity of particle size distribution of Co‐bipy for AP particle cutting leads to uncertainty of the final result. This situation can be avoided as much as possible by recrystallizing AP in the growth process of Co‐bipy, but it will increase the synthesis cost of solid propellant. Therefore, how to balance the combustion performance and overall cost of solid propellant will be the focus of future research.
2) Although Co‐bipy shows excellent catalytic behavior for the thermal decomposition of AP, its metal content is still very low. Therefore, it is extremely important to develop catalysts with high metal content to further improve the catalytic efficiency. However, this idea becomes more difficult due to the influence of ligands in complexes and coordination environment. Therefore, the development of functional ligands and the chemical design of a reasonable coordination environment determine the catalytic height of such materials.
3) This study has proved the structural impact of Co‐bipy on HTPB, a key component in solid propellants. In fact, this impact is likely to extend to other components. Although it sounds very attractive to open the active interface in the combustion process, the safety issues in the combustion process of propellants are still worth considering. In addition, the actual role of Co‐bipy catalysts in the combustion process of solid propellants still needs further verification, which is of great significance for the directional design of efficient combustion catalysts.

## 7 Experimental Section

### Materials

Cobalt nitrate hexahydrate (Co(NO_3_)_2_⋅6H_2_O), 4,4‐bipyridine (C_10_H_8_N_2_) with analytical grade were purchased from Shanghai McLin Biochemical Technology Co., Ltd and used without any further purification. Ammonium perchlorate were purchased from Yingkou Tianyuan Chemical Co., Ltd

### Syntheses of Co‐bipy

The Co‐bipy precursor was prepared as follows. Co(NO_3_)_2_⋅6H_2_O (0.10 g) was dispersed into 5 mL methanol to form a uniform aqueous Co(NO_3_)_2_ solution. 4,4‐bipyridine was dissolved into 5 mL absolute methanol. The Co(NO_3_)_2_ solution methanol solution was slowly dropped into the 4,4‐bipyridine solution by using a constant‐pressure drop funnel under magnetic stirring for 24 h. The obtained pink precipitate was centrifuged and washed thrice with methanol and placed in a vacuum drying oven at 60 °C for 12 h to obtain the Co‐bipy precursor powder.

### Syntheses of CoO/C

**Scheme 1:**
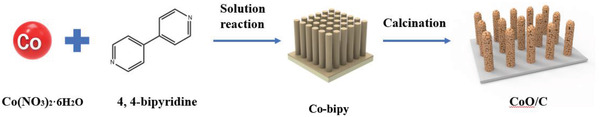
Schematic diagram of the synthesis process of Co‐bipy.

The obtained Co‐bipy precursor powder was placed in a tube furnace, and the temperature was increased to 400 °C at a heating rate of 2, 5, 5, 10, and 20 °C min^−1^, and kept for 1 h in N_2_ gas atmosphere. Uniformly dispersed CoO nanoparticles loaded on the N doped C base were obtained (**Scheme** **1**).

### Characterization

The powder X‐ray diffraction patterns of samples were examined via the X‐ray diffraction (XRD, Rigaku Miniflex600) using Cu‐k*α* rays (*λ* = 0.15418 nm) in a 2*θ* range of 3°–80° with a scan step width of 0.03°. The morphological properties of the materials were studied by field emission scanning electron microscope (FESEM) (UItra 55, Cari Zeiss, Germany). The FT‐IR spectra was obtained using the Nicolet 380 FT‐IR spectrophotometer (Thermo Fisher Nicolet, Waltham, MI, USA) in KBr pellet to investigate the chemical bonding of samples from 4000 to 400 cm^−1^. use ESCALAB250Xi XPS photoelectron spectrometer (XPS) instrument to test the element distribution and valence state of ZnCo‐ZIF surface were tested; The nitrogen adsorption–desorption isotherm of the sample was measured on a Quadra Chrome adsorber at 77 K. The total specific surface area of the sample was calculated by the Brunauer–Emmett–Teller method. Thermogravimetric analyzer (STA‐2500, NETZSCH) was carried out from room temperature to 800°C at a heating rate of 10 °C min^−1^ to check the thermal stability of the prepared Co‐bipy. The structure and decomposition products of Co‐bipy/AP with reaction temperature were monitored by TG‐IR and in situ IR.

### Catalytic Performance in the Thermal Decomposition of AP

The prepared samples were mixed with AP at a mass ratio of 3%, after mixed grinding. The prepared mixture was heated from 50 to 450 °C at a scanning speed of 10 °C min^−1^ to conduct DSC using the STA‐2500 DSC analyzer in N_2_ at 70mL min^−1^. The amount of the mixture used in each test was controlled within 0.5 to 1.5 mg .

## Conflict of Interest

The authors declare no conflict of interest.

## Supporting information

Supporting Information

## Data Availability

All data included in this study are available upon request by contact with the corresponding author.
