# Supplementary material for: In Situ Cutting of Ammonium Perchlorate Particles by Co‐Bipy “scalpel” for High Efficiency Thermal Decomposition
Source: Adv Sci (Weinh). 2022 Oct 30;9(35):2204109. doi: 10.1002/advs.202204109 (PMC9762298; doi:10.1002/advs.202204109)
Supplement: Supplementary file 1 — Supporting Information [file ADVS-9-2204109-s001.pdf]

ADVANCED SCIENCE
Open Access

Supporting Information

for *Adv. Sci.*, DOI 10.1002/advs.202204109

In Situ Cutting of Ammonium Perchlorate Particles by Co-Bipy "scalpel" for High Efficiency Thermal Decomposition

Peng Zhou, Siwei Zhang, Zhuoqun Ren, Xiaolin Tang, Kuan Zhang, Rui Zhou, Dan Wu, Jun Liao, Yifu Zhang and Chi Huang**

In-situ cutting of ammonium perchlorate particles by Co-bipy "scalpel" for high efficiency thermal decomposition

*Peng Zhou[12], Siwei Zhang[12], Zhuoqun Ren[12], Xiaolin Tang[12], Kuan Zhang[12], Rui Zhou[2], Dan Wu[2], Jun Liao[2], Yifu Zhang[12] *, Chi Huang[12] **

College of Chemistry and Molecular Sciences[1], Wuhan University, Wuhan, 430072; Research Center of Structure and Functional Materials, Hubei Key Laboratory of Aerospace Power Advanced Technology[2], Yichang 444200, China;

*Corresponding author: yfzhang@dlut.edu.cn; chihuang@whu.edu.cn

Keywords：Coordination polymer, ammonium Perchlorate, nano catalyst, structure self-transformation, catalytic mechanism

a

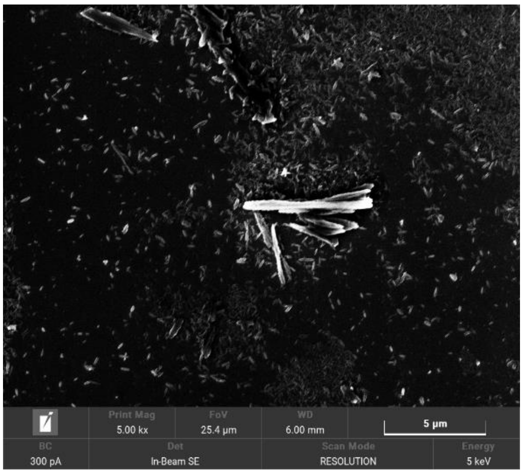

b

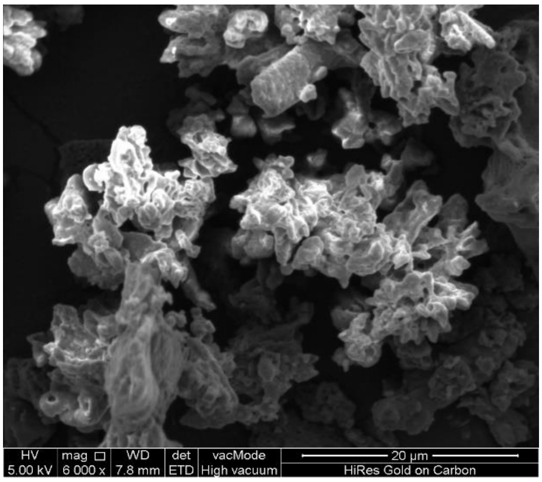

c

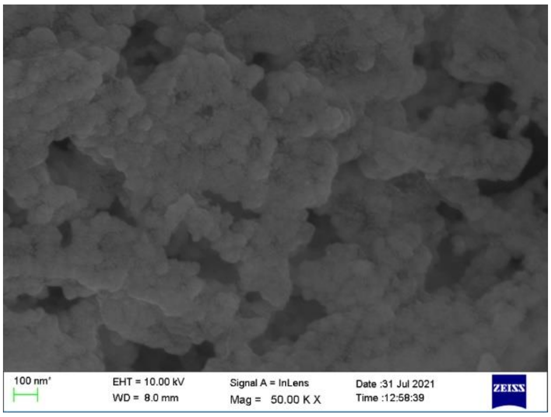

Fig. S1 SEM spectra of Co-bipy-2 (a), Co-bipy-2-600 (b) and Co-bipy-600 (c)

a

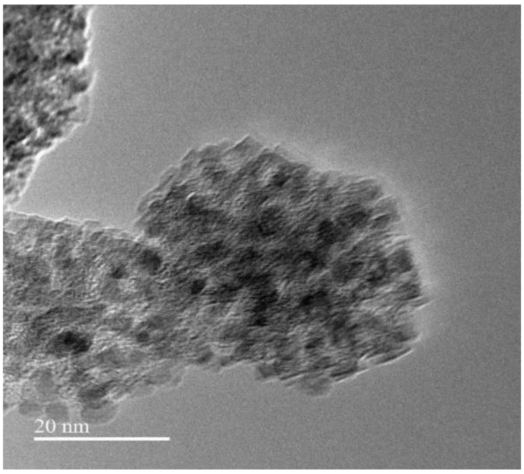

b

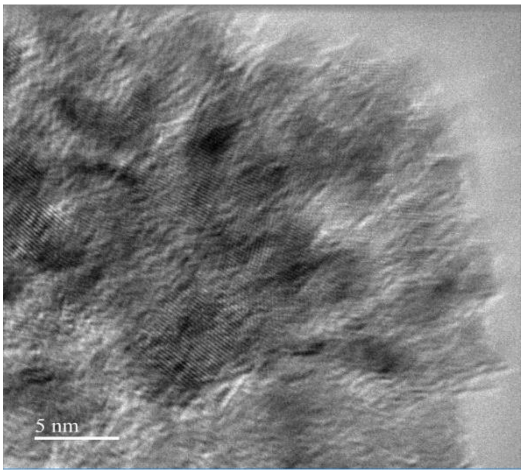

Fig. S2 TEM spectra of CoO/C.

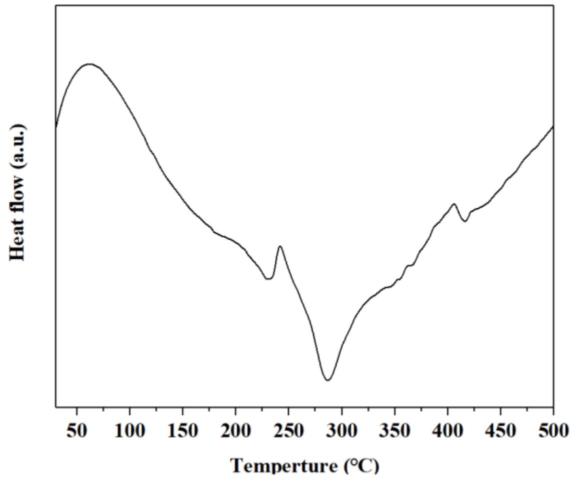

Fig. S3. DSC curves of NH_4NO_3 catalyzed thermal decomposition of AP.

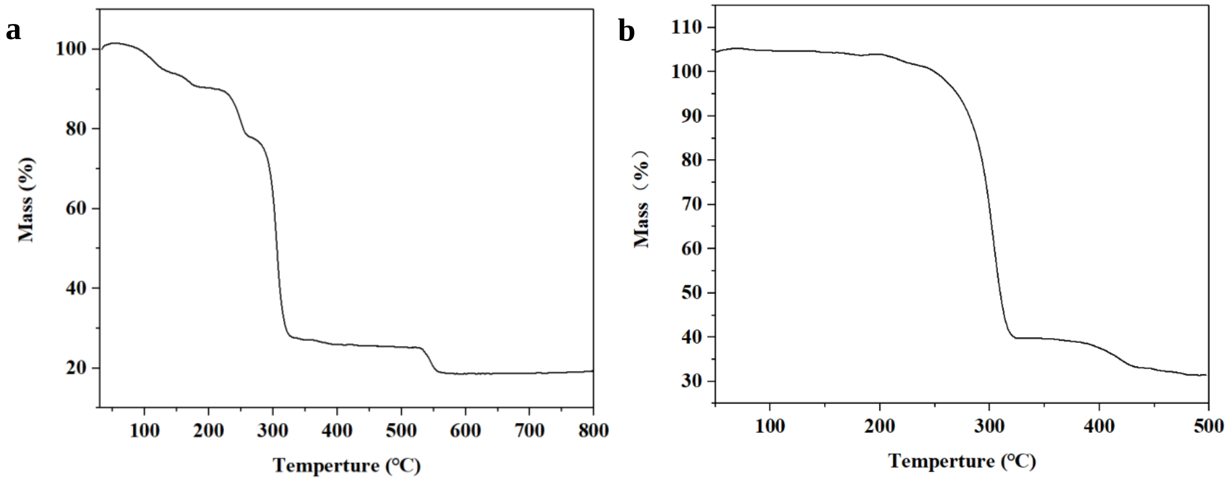

Fig. S4. TG curves of Co-bipy (a) and Co-bipy/AP (mass ratio is 1:1) (b).

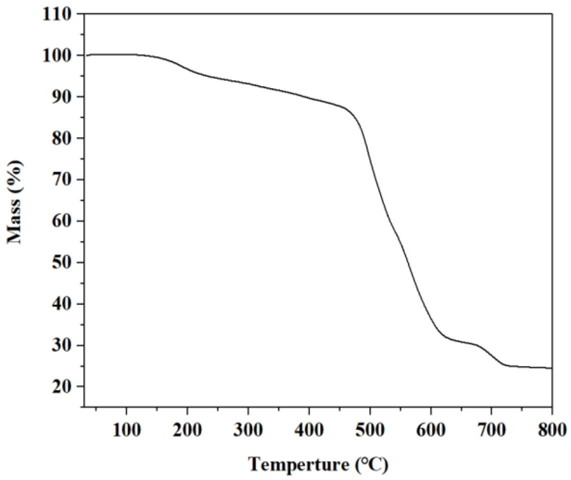

Fig. S5. TG curves of Co-bipy-2.

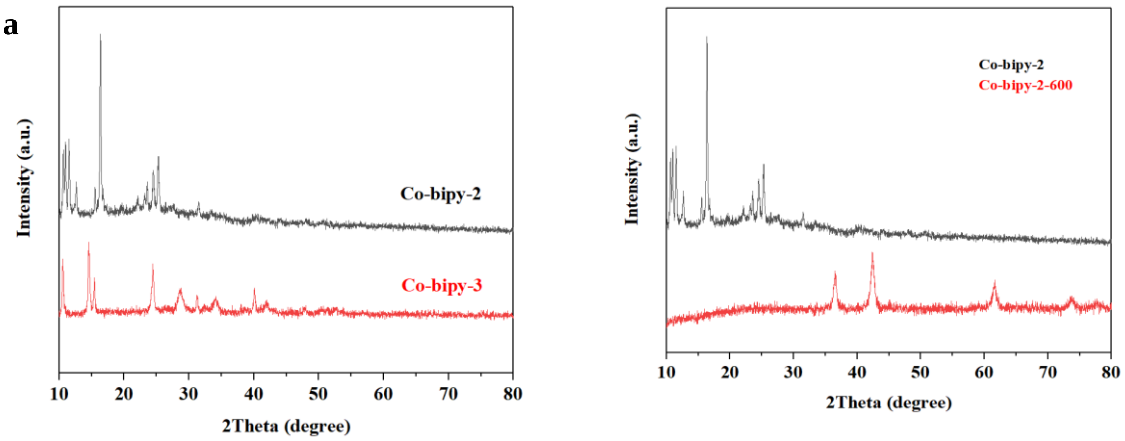

Fig. S6. XRD of Co-bipy-2/Co-bipy-3 (a) and Co-bipy-2-600 (b).

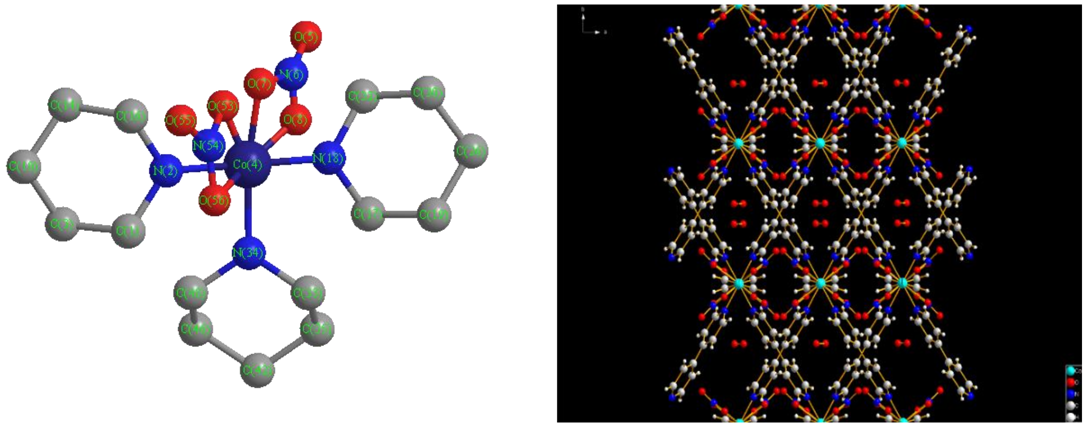

Fig. S7. Crystal structure of Co-bipy.

Crystal data show that three N from 4,4-bipyridyl and four O from nitrate around Co(II) form a distorted pentagonal bipyramid structure. In the axial direction, each Co(II) coordinates with the N of two 4,4-bipyridines to form a one-dimensional long chain structure. The pyridine rings in 4,4-bipyridines twist each other by 38.2 °, and the Co-N bond length in this direction is 2.1251 Å. As shown in the figure, such one-dimensional chain has two groups of marks in the crystal, one of which has an angle of 54.8 ° with the axis, and the other has an angle of -54.8 °. Two long chains are connected on the axis by a 4,4-bipyridine to form a three-dimensional

double-layer structure. The Co-N bond length on the axis is 2.1347 Å. In the framework structure formed by Co(II) and 4,4-bipyridine, each Co(II) forms a coordination structure with two NO_3^-, and the two Co-O bond lengths formed by each NO_3^- and Co(II) are 2.2261 Å and 2.3224 Å, respectively. H_2O molecules are filled between crystal structures, and there is no obvious bonding between H_2O molecules and frameworks. It can be seen that Co(II) and 4,4-bipyridine play a major role in forming the framework, and 4,4-bipyridine plays a role in forming one-dimensional long chains and bridging two long chains [32].

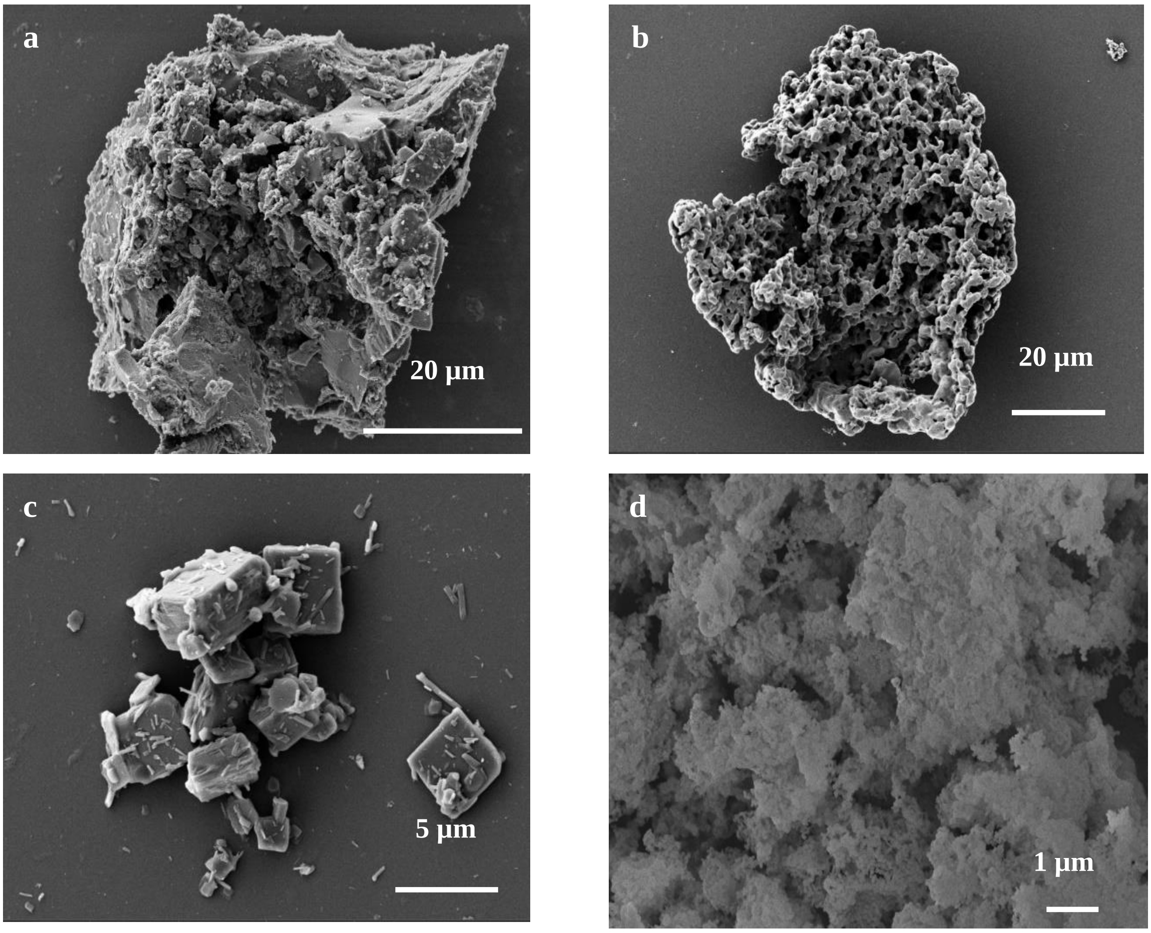

Fig. S8. SEM spectra of Zn-bipy and Ni-bipy before and after heat treatment (heating rate 1 °C min^{-1})

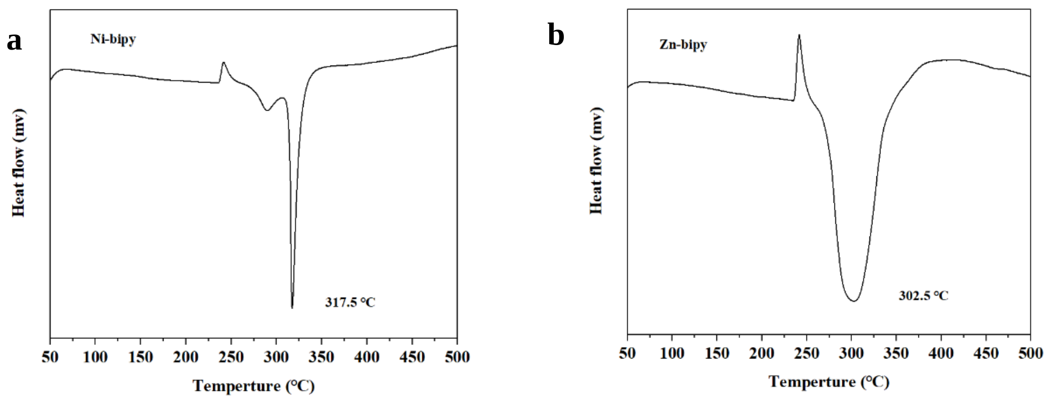

Fig. S9. Thermal Decomposition of AP Catalyzed by Ni-bipy (a) and Zn-bipy (b).
